# Performance of a Sensor-Based Health Monitoring System for the Detection of Clinical Mastitis in Dairy Cows

**DOI:** 10.3390/ani16213369

**Published:** 2026-10-26

**Authors:** Jule Marie Pries, Franziska Nankemann, Nicole Wente, Janina Schmidt, Doris Klocke, Volker Krömker

**Affiliations:** Department of Bioprocess Engineering and Microbiology, University of Applied Sciences and Arts Hannover, D-30453 Hannover, Germany; julep4@web.de (J.M.P.); franziska.nankemann@hs-hannover.de (F.N.);

**Keywords:** clinical mastitis, mastitis detection, automated health monitoring, reticuloruminal temperature, artificial intelligence, precision livestock farming

## Abstract

Mastitis is one of the most important production diseases in dairy farming and affects animal welfare, milk quality, and farm profitability. The use of automated health-monitoring systems has increased, and temperature-sensing reticular boluses are used to detect mastitis and other diseases or physiological responses. This study aimed to evaluate whether mastitis alerts generated by the smaXtec system are associated with subsequently diagnosed clinical mastitis under field conditions. An alert-based prospective case series was conducted on nine dairy farms in northern and western Germany. All cows receiving a smaXtec mastitis alert were included if a corresponding milk sample was available. Clinical examination, quarter milk sampling and microbiological analysis were performed. Among the 102 alerted cows, 40 were diagnosed with clinical mastitis within a 4-day observation period, with most of the cases being non-severe. This corresponded to a positive predictive value of 39.2%. We conclude that accelerometer- and temperature-based monitoring may support the detection of clinical mastitis, but this study showed limited diagnostic value under practical field conditions, when used as a stand-alone tool.

## Simple Summary

Mastitis is one of the most important diseases in dairy cows and negatively affects animal welfare, milk quality and farm profitability. Automated health monitoring systems are increasingly used to detect mastitis and other diseases. This study evaluated a commercially available monitoring system by smaXtec that uses algorithms and artificial intelligence to analyze data on body temperature and activity. The smaXtec system generates alerts for cows with suspected mastitis. We investigated 102 mastitis alerts from nine dairy farms in Germany after such an alert was generated and assessed whether the cows subsequently developed clinical mastitis, meaning mastitis with visible changes in the milk or udder. During the observation period, 40 of the 102 cows were diagnosed with clinical mastitis, while 62 cows, more than half of the examined cows, were not diagnosed with clinical mastitis. These findings suggest that these alerts may support the detection of clinical mastitis but should be followed by closer examination and never be used as the sole basis for treatment decisions.

## 1. Introduction

Mastitis is regarded as one of the most significant production diseases in dairy farming. It not only leads to economic losses due to reduced milk yield and increased costs, but also negatively affects animal welfare and milk quality [1]. With the growing adoption of automatic milking systems, automated detection of clinical mastitis (CM) is becoming a crucial component of milk quality assurance and animal welfare, as it may enable timely identification and, if necessary, treatment of affected animals [2]. Beyond its relevance for animal health and welfare, mastitis also affects antimicrobial use in dairy cattle and, consequently, antimicrobial stewardship. Antimicrobial treatment should be targeted to cases in which it is considered appropriate, as not all cases of clinical mastitis require antimicrobial therapy [3]. This is in line with the European approach to prudent antimicrobial use and antimicrobial stewardship as part of the One Health response to antimicrobial resistance [4]. The European Union (EU) aims to reduce antimicrobial sales for farmed animals and aquaculture by 50% by 2030, with antimicrobial treatment of udder infections contributing a substantial proportion of antimicrobial use in dairy cattle [3,4].

Dairy farmers and veterinarians have relied on body temperature measurements—particularly rectal temperature—as a very important parameter for assessing overall animal health, identifying and managing fever-related illnesses such as severe mastitis, pneumonia, and metritis, as well as metabolic or physiological changes in cows, such as estrus, heat stress, and the onset of calving [5].

In recent years, automated systems for monitoring core body temperature have emerged as an alternative method to support the early detection of both health disorders and key reproductive or metabolic events [6]. Compared with manual measurement, automated temperature monitoring allows more frequent data collection without direct handling of the cow, minimizing stress and time-intensive labor [7].

Possible locations for the automated measurement of body temperature include the vagina [8,9], the intraperitoneal cavity [10,11], the tympanum [5,12], the udder [10], the udder surface [13] and the rumen [7,14]. Among these, some methods, such as monitoring the body temperature inside the vagina or the external ear canal, have shown promising accuracy but seemed to cause infections and could only be used for a limited time period [11,12].

Building on these developments, the measurement of reticuloruminal temperature (RTT) has gained attention as a possible approach for the monitoring of the overall health status and the detection of various production diseases in dairy cows [7,9]. In particular, RTT monitoring has been associated with the early detection of mastitis and pneumonia [7]. Ammer et al. concluded that, despite the considerable variation in reticular temperature measurements and various influencing factors, reticular temperature monitoring may be a useful approach for monitoring dairy cows [9]. To enable automatic RTT measurement, temperature-sensing reticular boli are used, allowing RTT monitoring at regular intervals (e.g., every ten minutes) [7,9].

Bewley et al. [15] found a correlation between rectal temperature and RTT of r = 0.645, which they classified as a strong correlation. Sievers et al. [16] found a correlation of r = 0.92 and concluded that changes in body temperature could be reliably documented by an intraruminal device. In a 2001 study, the peritoneal cavity and the lower rumen were found to be effective sites for measuring core body temperature [11]. One commercial system that utilizes this principle is the smaXtec health monitoring system, which uses an indwelling bolus placed in the reticulum to measure temperature, movement and rumination activity, as well as to estimate water intake. According to the manufacturer, the collected data are transferred to a cloud and then analyzed using artificial intelligence (AI) to identify deviations from the individual cow’s normal temperature baseline. When such deviations occur, the system supposedly generates a specific alert, referred to as a ‘mastitis alert’ in the following, for animals suspected of having mastitis [17,18].

Numerous factors influence RTT. For example, the water intake influences RTT dramatically. Particularly large volumes of cold water can decrease RTT substantially for at least 3 h [6]. Additional factors include ambient temperature, milk yield, breed and feeding activity, as decreased food intake can result in reduced rumen fermentation [19]. Body temperature in general is further influenced by time of day, months, age, ambient temperature, temperature-humidity index, overall health, level of activity, estrus, feeding habits, excitement, parity and lactation state [5,6,8,10,20].

Given the wide range of factors influencing RTT, understanding the physiological basis of fever is essential to distinguish fever-related temperature changes from those associated with other factors.

Roth and Blatteis describe fever as “a cardinal symptom of infectious or inflammatory insults”, though they also note that fever can occur from noninfectious causes [21]. It is important to distinguish fever from hyperthermia. In hyperthermia, a rise in core body temperature results from altered thermoregulation, whereas fever is a coordinated, brain-regulated component of the host’s nonspecific defense mechanisms against pathogenic threats [21,22,23]. Fever results from a pyrogen-induced elevation of the hypothalamic set point for core temperature. Exogenous pyrogens, such as bacteria and bacterial components, trigger the release of endogenous pyrogens [21]. These stimulate the production of prostaglandin E2, which is believed to be a key mediator in fever development [21,24]. Important pyrogens regarding mastitis include lipopolysaccharide (LPS), a cell wall component of Gram-negative bacteria such as *Escherichia coli* (*E. coli*) [21]. In Gram-positive bacteria, lipoteichoic acid (LTA) is an important pyrogenic component [25]. Wellnitz and Bruckmaier showed that LPS from *E. coli* results in a more extensive immune reaction with regard to the transfer of blood components into milk than LTA from *Staphylococcus aureus*. These differences can be explained by diverse patterns of the opening of the blood–milk barrier in various mastitis-causing pathogens [26,27,28].

The smaXtec system also considers activity and movement when generating mastitis alerts, although the manufacturer does not disclose their individual weights. Behavioral parameters can be relevant for mastitis detection, as clinical mastitis is considered to be a painful condition [29,30]. Pain related to mastitis may influence behaviors such as feeding and ruminating, walking and lying down, the position of the tail, and the reaction to forestripping [31,32]. Several studies have specifically reported alterations in rumination activity and movement in cows with mastitis. An experimental study showed that rumen motility first decreased and then increased after infusion with LPS [32]. Kester et al. [33] observed a reduced number of total steps a day in cows with experimentally induced *Streptococcus uberis* mastitis. In contrast, a study examining cows with naturally occurring mastitis found increased steps compared with healthy cows [31].

Previous studies have demonstrated associations between individual sensor-derived parameters, such as RTT, activity, and rumination, and mastitis [7,9,14,32,34]. Nevertheless, findings from studies that investigate individual sensor parameters or experimentally induced mastitis cannot necessarily be generalized to the performance of commercially available, multifactorial, AI-based mastitis alerts under routine field conditions.

Therefore, this study aimed to evaluate the association between mastitis alerts and subsequently diagnosed cases of CM in dairy cows with a focus on the positive predictive value.

## 2. Materials and Methods

### 2.1. Study Design and Animal Population

This alert-based prospective case series was conducted from January 2025 to November 2025 on nine dairy farms located in northern and western Germany, with animals enrolled at the time of sensor alerts and followed for four days. Herd sizes ranged from 230 to 545 lactating Holstein-Friesian cows. Average annual milk yield per cow at the herd level ranged from 10,200 to 13,510 kg. Bulk milk somatic cell counts varied from 80,000 to 276,000 cells/mL. The reported incidence rate of clinically treated mastitis cases per 365 cow-days at risk ranged from 2.7% to 27.9% with a mean mastitis rate of 14.7%. Eight of the nine farms used conventional milking systems, while one farm operated an automatic milking system. All participating farms, except one, reported using mastitis alerts as a trigger to examine and possibly treat the affected cow, with seven using it as their primary detection tool. All nine applied at least one additional method to verify mastitis cases alerted by smaXtec. Six farms used the California Mastitis Test to indirectly estimate the somatic cell count and to visually assess milk abnormalities, such as flakes. Two farms used milk pH values to assess the integrity of the blood-milk barrier. Another two farms relied on electric conductivity measurements from the milking system and only one farm reported applying visual inspection as a verification method.

All participating farms used the smaXtec systems (© smaXtec animal care GmbH, Graz, Austria) for continuous measurement of reticular temperature, which constituted the primary inclusion criterion. SmaXtec provided contact information for eight of the nine participating farms, while the remaining farm was sourced through a personal contact. Additionally, farms had to be located in northwest Germany and consent to participate in the study, which entailed additional workload for farm personnel. From these farms, every cow receiving a mastitis alert via the smaXtec system was included in the study, provided that a corresponding milk sample was available for analysis.

Assuming an expected positive predictive value (PPV) of 80%, the sample size was calculated to demonstrate a PPV significantly greater than 60% under the null hypothesis. Using a one-sided significance level of α = 0.05 and a power of at least 80%, a minimum of 36 test-positive cases were required based on an exact binomial test. As the observed PPV during the study was lower than expected, the study period was extended to increase the sample size.

Detailed information on the algorithms and artificial intelligence used by the manufacturer to generate mastitis alerts was not available to the authors, as this information is proprietary to © smaXtec animal care GmbH. Consequently, this study could not describe the specific technical details of the AI-based alert generation.

### 2.2. Sample Collection, Laboratory Analysis and Data Acquisition

Upon receiving an alert from the smaXtec system, indicating a potential mastitis case, a milk sample from every lactating udder quarter was aseptically collected by farm personnel. Staff were trained to detect CM and instructed in proper aseptic sampling technique. Ly20, a boric acid-based preserving agent, was used in test tubes [35]. Samples were cooled and promptly sent to the University of Applied Sciences and Arts Hannover (Germany), where the microbiological examinations were performed in accordance with the guidelines of the German Veterinary Medical Association [36]. Somatic cell counts (SCC) were assessed using flow cytometry with the SomaScope™ Smart system (Delta Instruments B.B., Drachten, The Netherlands). At the start of the microbiological examination, 10 μL of each milk sample was streaked on one quadrant of an esculin blood agar plate (5% defibrinated sheep blood, Oxoid Deutschland GmbH, Wesel, Germany) and incubated at 37 °C, with colony growth evaluated after 24 and 48 h. Growing colonies were identified regarding their genus and species via matrix-assisted laser desorption ionisation time-of-flight (MALDI-TOF, Bruker Daltonics, microflex LT/SHsmart, MBT Compass Library, V8, Bremen, Germany). Based on cell morphology, yeasts, moulds and Prototheca were differentiated and therefore, Gram staining was performed. Milk samples were classified as microbiologically positive if at least five colonies of the same environmental-associated mastitis-causing pathogen were detected. However, a single colony of *Staphylococcus aureus*, *Streptococcus agalactiae*, *Trueperella pyogenes* or *Streptococcus dysgalactiae* was sufficient to consider the sample positive. If more than two distinct pathogens were present on one plate, the sample was categorized as contaminated.

Farmers agreed to perform clinical examinations and milk sampling as soon as possible after every occurring mastitis alert, following their daily routines. Farm personnel also completed a standardized questionnaire that included basic information about the cow (e.g., lactation number, days in milk) and more detailed information about the suspected mastitis event and observed clinical signs, such as local signs of udder inflammation, changes in milk and systemic signs of severe mastitis, like fever (≥39.5 °C) and lack of appetite. In addition, treatment measures on the day of sampling and other anomalies concerning udder, milk and milking process were documented. Any treatment initiated was not influenced by us and was solely up to the farm’s procedures.

The PPV was calculated as the proportion of mastitis-alert events associated with clinical mastitis during the observation period among all mastitis alert-events. The 95% confidence interval (CI) was calculated using the Wilson score method.

### 2.3. Definition of Clinical Mastitis and Observation Period

Clinical mastitis cases were classified as non-severe or severe according to international definitions. Cases were considered severe when grave systemic symptoms such as fever, anorexia, or recumbency were observed. Non-severe cases were characterized by local alterations, such as abnormal milk or visible inflammation of the affected udder quarter, without evidence of systemic involvement, and encompassed both mild and moderate clinical mastitis [37].

The observation period was defined as the day of milk sample collection (day 0), followed by four subsequent days, as the manufacturer states that changes in body temperature associated with pathogenic infection occur up to four days before the onset of external clinical signs [18]. After these four days, we contacted the farms to document whether CM occurred subsequently or whether any non-severe CM cases detected during the initial clinical examination worsened to severe CM.

## 3. Results

A total of 103 mastitis alerts were reported and documented. One shipment with milk samples was lost during transport and therefore could not be included in the analysis, resulting in 102 mastitis alerts included in the study. These alerts derived from 101 individual cows, as one cow received two mastitis alerts several months apart. Both alerts were considered separate events and were included independently in the analysis. Across the 102 alert events, the mean lactation number was 3.3 (range: 1–8), and the mean number of days in milk was 125.2 days (range 9–463). Included mastitis alerts per farm ranged between 3 and 23, with a mean of 11.3 alerts per participating farm, not necessarily corresponding with a relatively higher or lower incidence rate of clinically treated mastitis cases per 365 cow-days at risk reported by the farms.

Of the 102 mastitis alerts included, 21 were diagnosed with CM on the day of milk sample collection. Among these cases, 18 were classified as non-severe and three as severe. An additional 19 cows were diagnosed with CM within four days after sample collection, of which 14 were classified as non-severe and five as severe (Table 1). In 35 cows, only one udder quarter was affected. Five cows showed clinical mastitis in two udder quarters. In total, there were 36 udder quarters diagnosed with non-severe CM and nine udder quarters diagnosed with severe CM. The remaining 62 cows, or 355 udder quarters, were not diagnosed with CM during the observation period. Eight cows had only three lactating udder quarters, and consequently only three quarter milk samples, which explains why only 400 milk samples are available.

### 3.1. Positive Predictive Value

The study design did not allow for a complete ascertainment of all clinically mastitic and all healthy cows in the examined populations. Therefore, sensitivity and specificity of the smaXtec system could not be estimated. Consequently, diagnostic performance was assessed using the PPV according to the following formula:

$$PPV=\;\frac{Number\;of\;True\;Positives}{Number\;of\;True\;Positives+Number\;of\;False\;Positives}$$

Of the 102 cows that triggered a mastitis alert, 40 cows were diagnosed with CM during the observation period and were therefore classified as true positives. The remaining 62 alerted cows did not develop clinical mastitis within the observation period and were classified as false positives. Accordingly, the PPV of the smaXtec mastitis alert for detecting CM during the observation period was 39.2% (40/102; Table 1; 95% CI: 30.3–48.9). PPV ranged per farm from 0% to 100% (Table 2).

### 3.2. On-Farm Responses to Mastitis Alerts

Among cows diagnosed with CM within the observation period, 15% were treated with a non-steroidal anti-inflammatory drug (NSAID) only. The remaining 85% with CM received antibiotics as well as NSAID therapy. Among cows not diagnosed with CM, 51.6% were treated with NSAIDs only, while 11.3% received antibiotics, and 8.1% also received NSAID treatment (Table 3).

### 3.3. Microbiological Findings

A total of 400 quarter milk samples were examined microbiologically. Samples showing no bacterial growth were most common among quarters without clinical mastitis. In samples from udder quarters with non-severe CM, Gram-positive bacteria were most frequently detected, whereas Gram-negative bacteria were more frequently detected in samples from quarters with severe CM (Table 4). The most frequently detected pathogens in udder quarters with CM were *Escherichia coli* (*E. coli*), *Streptococcus* spp. and mixed bacterial species (Table 5). Pathogens that are commonly assigned to the group of coliform bacteria (in this case *E. coli*, *Klebsiella* spp., *Raoultella* spp. and *Hafnia alvei*), were detected in 47 quarters out of all samples (11.8%), in 32 samples of udder quarters without clinical mastitis (9%), in 11 out of 36 udder quarters with non-severe CM (30.6%) and in 4 out of 9 udder quarters with severe CM (44.4%) (Table 6).

## 4. Discussion

### 4.1. Study Objective

This study aimed to evaluate whether mastitis alerts generated by an AI analyzing data from temperature-sensing reticulum boli and accelerometers are associated with the occurrence of CM under field conditions.

### 4.2. Study Design and Limitations of the Study

We conducted an alert-based prospective case series, which did not include a control group. Since the cows were not examined daily for signs of CM, we were not able to assess sensitivity, specificity or the negative predictive value, but rather estimated the PPV, which is a key figure in the practical use of automated health monitoring systems. The sample size of 102 mastitis alerts may seem small but is sufficient to evaluate system performance. The treatment was not dictated by us, and it could be hypothesized that a treatment at the time of the mastitis alert could prevent the occurrence of CM or rather the transition from a non-severe CM to a severe CM. Though in this case a disproportionately small number of severe CM cases might be expected, which is not the case in this dataset, compared to the study done by Schmenger and Krömker in 2020 [38]. It is possible that treatment with either AB, NSAID, or both, administered in 79 cases, influenced the occurrence of clinical findings in the four days following milk sampling to such an extent that no further clinical signs appeared. However, German Guidelines [39] limit treatment of subclinical mastitis during lactation to special cases involving infections caused by rapidly spreading microorganisms. Therefore, a microbiological examination would have been required for treatment to comply with the guidelines. All enrolled cows originated from only nine high-producing Holstein-Friesian herds located in northern and western Germany, which restricts extrapolation of the results to other herd types, breeds, regions and management systems. Moreover, the distribution of mastitis severity and causative pathogens in our alerted cases may not reflect the overall mastitis and pathogen profile of other dairy herds, so that the performance of the system could differ in populations with substantially different disease- and pathogen patterns. One potential source of selection bias is that smaXtec provided contact information for eight of the nine participating farms. However, smaXtec was not involved in the selection of individual cows, sample collection, or clinical evaluation. Nevertheless, farms recruited through the manufacturer may not represent the overall population of farms using the system, which could limit the generalizability of the results. The time of year in which most of the samples were taken may also be a possible influence factor on the severity of CM and on bacteriological findings in the quarter milk samples. Furthermore, it has to be addressed that the clinical examination, the milk sampling and the completion of the questionnaire did not necessarily take place immediately after generation of the mastitis alert. The timing depended heavily on the time management and personnel availability, which may vary not only between farms but also by season and time of day. Although this is a limitation, since standardized timing was not possible in this study, it also reflects the normal conditions under which health monitoring systems are applied in practice. Finally, the data collected through the questionnaires relied on self-reported information by the farmers, meaning that inaccurate reporting or incomplete documentation may have affected the reliability of the results. In addition, as access to the smaXtec accounts was not available to us for all farms, alerts had to be reported by the farmers themselves, and some alerts may therefore have gone unreported. Despite these limitations, this study provides valuable groundwork for future research by offering a first step towards evaluating the performance of this system with regard to the detection of CM in dairy cows.

### 4.3. Diagnostic Performance

The PPV observed in this study indicates that a substantial proportion of alerts were not associated with clinically diagnosed mastitis. Nevertheless, it must be considered that this value is highly dependent on the prevalence of the disease, meaning that even highly accurate systems may produce a considerable proportion of false-positive alerts in low-prevalence settings [40]. However, the prevalence of clinical mastitis among the study population could not be determined in the present study. Only the incidence rate of clinically treated mastitis cases per 365 cow-days at risk reported by the farms were available, with considerable variation between farms. Therefore, it cannot be determined whether the prevalence of mastitis in the study population contributed to the observed PPV. Several factors may have contributed to the relatively low PPV. The sensor-derived parameters used by the system are not specific to mastitis and may be influenced by various physiological, environmental, and behavioral factors [6,19,31,32,33]. Consequently, deviations in these parameters may trigger an alert without the presence of clinical mastitis. We focused on clinical mastitis in this study, whereas the smaXtec system may potentially generate alerts in response to physiological changes associated with subclinical mastitis. In the present analysis, such alerts would have been classified as false positives if no clinical mastitis was diagnosed during the observation period. As subclinical mastitis was not specifically investigated, this possibility cannot be confirmed based on the present data.

### 4.4. Associations Between Mastitis Severity and Temperature Changes

When examining the connection between fever and mastitis, it should be considered that, according to the International Dairy Federation (IDF), only severe CM is associated with systemic effects like fever and mild or moderate CM typically involve only local changes in the milk and udder [37]. In this study, 80% of all detected cases were classified as non-severe, whereas the remaining 20% were categorized as severe CM. In comparison to the study by Schmenger and Krömker [38], this shows an overrepresentation of severe CM, which is in line with the expectations regarding the connection between mastitis severity and body temperature. This may also indicate that the treatment of cows showing an increased RTT with NSAIDs and/or antibiotics did not necessarily prevent the development of severe CM in these cases. However, since the absolute number of severe mastitis cases was relatively small, despite being overrepresented in this study, these findings should be interpreted with caution, and a definite effect cannot be determined through this study. Preferential detection of severe cases may still be clinically relevant, since severe CM is associated with greater tissue damage, systemic impairment, and more pronounced economic losses. In 5 of 8 severe CM cases in this study, a mastitis alert was issued before the farmer could diagnose the mastitis.

The findings of a prospective case–control study conducted on a Colorado dairy farm [7] support the plausibility of using reticuloruminal temperature for mastitis detection, as cows with CM showed markedly higher odds of exhibiting an increase in reticular temperature rise above their individual baseline compared with periods in which the same cows were not diagnosed with mastitis. By contrast to the smaXtec system used in this study, Adams and colleagues calculated a clear cut-off point at which a mastitis alert should be generated to allow for the best system performance [7].

### 4.5. Pathogen-Specific Differences in Temperature Response

The most common mastitis-causing pathogens for non-severe and severe CM in our study were *E. coli* and coliform bacteria in general. Overall, coliforms occurred at a substantially higher proportion than reported by Schmenger and Krömker [38], who found this pathogen group in 11.6% of mastitic milk samples. Differences between the examined cow populations may partly explain this discrepancy, despite the comparable geographic regions. Another possible explanatory approach may relate to the detection mechanism of the smaXtec system. Based on the strong pyrogenic activity of LPS described by Bruckmaier and Wellnitz [28], it could be hypothesized that mastitis caused by coliform pathogens may be more likely to result in a mastitis alert. The findings raise the possibility that pathogen-specific characteristics may influence temperature responses and, consequently, the likelihood of generating a mastitis alert.

A possible explanation could be diverse patterns for opening the blood-milk barrier and differences regarding the exogenous pyrogens leading to different amounts of endogenous pyrogens being released, and to differences in temperature changes and fever when looking at mastitis caused by different pathogens. This hypothesis is supported by the fact that in the study done by Bruckmaier and Wellnitz [28], only the cows challenged with LPS showed fever, while the rectal temperature of cows challenged with LTA remained normal. In contrast, Rambeaud et al. [41] demonstrated that CM experimentally induced with *Streptococcus uberis* was accompanied by an increase in rectal temperature in all nine infected cows. However, the timing of the temperature peak varied considerably among individuals during the course of infection. Another study that experimentally induced mastitis with *Streptococcus uberis* found an increased RTT one day after the intramammary challenge and showed a relation between higher bacterial counts and increased sensitivity for identifying intramammary infections with RTT [14]. This suggests a correlation between body temperature or clinical signs in general and bacterial density, which Rambeaud et al. [41] also reported. With this in mind, it needs to be considered that differences between experimentally induced and naturally occurring mastitis, for example, the method of exposure and challenge dose, can lead to differences in bacterial growth and subsequently alter the accuracy of mastitis alerts [14,42].

### 4.6. Comparison with Other Sensor-Based Detection Approaches

Since the smaXtec system does not rely solely on RTT, other factors must be considered. Movement and rumination activity seem to be useful parameters for identifying cows with clinical mastitis, as shown by several studies [32,33,34,43]. An interesting finding in the study of Stangaferro and colleagues [34] is that the performance of automated health-monitoring systems using rumination time and physical activity as reference factors is effective in cows with CM caused by an intramammary infection with *E. coli* but is only moderate for CM cases caused by pathogens other than E. coli. This is consistent with our considerations regarding early diagnosis of CM caused by different pathogens. Furthermore, these factors may enable algorithms and artificial intelligence to differentiate between clinical mastitis and other diseases, for example, lameness [44], or possibly events like high outdoor temperatures.

### 4.7. Implications for Treatment and Antibiotic Use

The treatment of CM is an essential but complex aspect of dairy health management. In this study, the majority of non-severe mastitis cases were treated with NSAIDs in combination with antibiotics, administered either locally or systemically. Nankemann and colleagues [45] reported that intramammary infections with *E. coli* do not benefit from antibiotic therapy in terms of bacteriological cure (BC). Another factor influencing BC is the mastitis history, as the likelihood of BC significantly decreases if cows were previously affected by clinical mastitis more than once in the current lactation [46]. In addition, cows with a SCC exceeding 700,000 cells/mL on at least three occasions are considered unsuitable candidates for antibiotic therapy. These factors, along with the use of a quick diagnostic test system when available, should be considered when treating non-severe CM in order to reduce unnecessary antibiotic use and limit the development of antimicrobial resistance [47]. According to the German Veterinary Medicinal Products Act [48], animal care personnel are permitted to administer antimicrobial medicinal products only when prescribed by a veterinarian or when acting under a valid veterinary treatment instruction. In this context, the comparatively high rate of antibiotic treatment observed in the present study should be interpreted with caution. In contrast, severe CM cases, including those caused by *E. coli*, justify treatment with systemic antibiotics due to the risk of life-threatening complications [45,47]. We documented 7 cases out of the 62 cases without CM in which cows were treated with antibiotics, despite showing no clinical symptoms. Especially in these cases, it is possible that the treatment influenced the development of clinical symptoms, prevented the occurrence of clinical mastitis, and consequently the PPV.

### 4.8. Role and Limitations of Artificial Intelligence

Using AI to analyze the collected data, presents another significant challenge: the so-called “black-box” nature [49]. This term describes that a human will not be able to understand how AI algorithms arrive at a response. While using AI algorithms promises to identify aspects of data that are not immediately apparent to a human observer or to a non-AI algorithm, this may reduce accountability, transparency and possibly trust in the system [49,50]. This issue is relevant to the present study, as the smaXtec mastitis alerts are generated by a proprietary AI-based algorithm. As mentioned above, we did not have access to the specific algorithm structure, thresholds, weighting of the individual parameters or training data underlying the alerts. Consequently, we cannnot determine how the sensor-derived parameters contributed to an individual mastitis alert or explain why an alert was generated in cases where no clinical mastitis was subsequently diagnosed.

Although discussion of AI and the development of “explainable AI” is already well-advanced, veterinary medicine lacks concepts such as informed consent and regulatory frameworks [50,51]. In practice, this may result in undetected mistakes, unreflective treatment decisions, or a loss of trust in the system due to lack of transparency.

### 4.9. Future Research

Further studies, including appropriate control groups, are required to assess sensitivity, specificity, and negative predictive value of automated health monitoring systems using reticuloruminal temperature to detect mastitis. In addition, future research should investigate the ability of such systems to detect subclinical mastitis and the pathogen distribution of the correctly detected mastitis cases. Standardized study designs and more diverse populations would help to validate the applicability of these systems across different herd types and management conditions.

## 5. Conclusions

In this field-based pilot study, the majority of mastitis alerts were not associated with a diagnosis of clinical mastitis within a four-day observation period. The observed distribution of clinical mastitis cases raises the question of whether the AI-based system may detect severe CM more readily than non-severe CM and how pathogen-specific characteristics may influence the probability of generating a mastitis alert. These hypotheses are exploratory and require confirmation in more extensive studies. The findings indicate that data from temperature-sensing reticulum boli and accelerometers, which have been analyzed by AI, may support the detection of CM, but their diagnostic value under practical conditions appears limited when used as a standalone tool. Therefore, mastitis alerts should never be the sole reason for treatment of CM in dairy cows.

## Figures and Tables

**Table 1:** Positive predictive values of smaXtec mastitis alerts for detecting non-severe and severe clinical mastitis at 0 and 4 days after quarter milk sampling.

Degree of Severity	Day 0	Days 1–4	Total
Non-severe CM	18/102 (17.6%)	14/102 (13.7%)	32/102 (31.4%)
Severe CM	3/102 (2.9%)	5/102 (4.9%)	8/102 (7.8%)
Total	21/102 (20.6%)	19/102 (18.6%)	40/102 (39.2%)

Note. Values represent the number of cows diagnosed with clinical mastitis relative to the total number of cows that triggered a smaXtec mastitis alert (n = 102). Percentages indicate the positive predictive value of the alert for detection of clinical mastitis within the specified time period. Day 0 refers to the day of sampling as soon as possible after the alert generation and Day 1–4 to the four subsequent days. CM = clinical mastitis.

**Table 2:** Number of included mastitis alerts, incidence rate of clinically treated mastitis cases per 365 cow-days at risk and positive predictive value per farm.

Included Alerts, n	Incidence Rate (%)	PPV (%)
3	8.6	0
7	10.5	85.7
8	2.7	0
9	14.3	66.7
9	15.1	100
10	27.9	40
16	22	18.8
17	15.7	5.9
23	16	47.8

Note. Included alerts are all alerts per farm that were included in the study. Incidence rate = incidence rate of clinically treated mastitis cases per 365 cow-days at risk. PPV = positive predictive value.

**Table 3:** Treatment administered to cows with non-severe or severe and without clinical mastitis following a smaXtec mastitis alert (n = 102).

Clinical Status	Only AB, n (%)	Only NSAID, n (%)	AB and NSAID, n (%)	No Treatment, n (%)	Total
No CM	2 (3.2%)	32 (51.6%)	5 (8.1%)	23 (37.1%)	62
Non-severe CM	0 (0%)	5 (15.6%)	27 (84.4%)	0 (0%)	32
Severe CM	0 (0%)	1 (12.5%)	7 (87.5%)	0 (0%)	8
Total	2	38	39	23	102

Note. AB = antibiotic treatment; NSAID = non-steroidal anti-inflammatory drug; CM = clinical mastitis. Percentages represent the proportion of cows within each clinical category (row).

**Table 4:** Distribution of microbial findings in quarter milk samples according to clinical mastitis severity (N = 400).

Degree of Severity	No Bacterial Growth	Gram-Negative Bacteria	Gram-Positive Bacteria	Mixed Bacterial Species	Total
No CM	180 (50.7%)	66 (18.6%)	105 (29.6%)	4 (1.1%)	355
Non-severe CM	5 (13.9%)	12 (33.3%)	18 (50.0%)	1 (2.8%)	36
Severe CM	2 (22.2%)	4 (44.4%)	1 (11.1%)	2 (22.2%)	9
Total	187 (46.8%)	82 (20.5%)	124 (31.0%)	7 (1.8%)	400

Note. CM = clinical mastitis. Values represent the number of quarter milk samples with the respective microbiological findings. Percentages were calculated within each clinical category.

**Table 5:** Three most frequent microbiological findings after a smaXtec mastitis alert according to mastitis severity.

Total, n (%)	No CM, n (%)	Non-Severe CM, n (%)	Severe CM, n (%)
No bacterial growth, 187 (46.8%)	No bacterial growth, 180 (50.7%)	*E. coli*, 9 (25.0%)	*E. coli*, 2 (22.2%)
NaS, 77 (19.3%)	NaS, 75 (21.1%)	*Streptococcus* spp., 8 (22.2%)	Mixed bacterial species, 2 (22.2%)
*Pseudomonas* spp., 28 (7.0%)	*Pseudomonas* spp., 27 (7.6%)	No bacterial growth, 5 (13.9%)	No bacterial growth, 2 (22.2%)

Note. CM = clinical mastitis, NaS = non-aureus staphylococci, *E. coli* = *Escherichia coli*; Percentages in the columns “no CM”, “non-severe CM” and “severe CM” are calculated within each respective clinical group.

**Table 6:** Distribution of bacterial isolates in quarter milk samples by clinical mastitis severity (N = 400).

Microbiological Findings	Total, n (%)	No CM, n (%)	Non-Severe CM, n (%)	Severe CM, n (%)
No bacterial growth	187 (46.8%)	180 (50.7%)	5 (13.9%)	2 (22.2%)
NaS	77 (19.3%)	75 (21.1%)	2 (5.6%)	0 (0%)
*Pseudomonas* spp.	28 (7.0%)	27 (7.6%)	1 (2.8%)	0 (0%)
*E. coli*	24 (6.0%)	13 (3.7%)	9 (25.0%)	2 (22.2%)
*Streptococcus* spp.	21 (5.3%)	13 (3.7%)	8 (22.2%)	0 (0%)
Coryneform bacteria	11 (2.8%)	7 (2.0%)	4 (11.1%)	0 (0%)
*Klebsiella* spp.	10 (2.5%)	9 (2.5%)	0 (0%)	1 (11.1%)
*Staphylococcus aureus*	8 (2.0%)	5 (1.4%)	2 (5.6%)	1 (11.1%)
*Raoultella* spp.	7 (1.8%)	7 (2.0%)	0 (0%)	0 (0%)
Mixed bacterial species	7 (1.8%)	4 (1.1%)	1 (2.8%)	2 (22.2%)
*Hafnia alvei*	6 (1.5%)	3 (0.8%)	2 (5.6%)	1 (11.1%)
*Pantoea agglomerans*	5 (1.3%)	5 (1.4%)	0 (0%)	0 (0%)
*Enterococcus* spp.	2 (0.5%)	2 (0.6%)	0 (0%)	0 (0%)
*Microbacterium maritypicum*	1 (0.3%)	1 (0.3%)	0 (0%)	0 (0%)
*Acinetobacter johnsonii*	1 (0.3%)	1 (0.3%)	0 (0%)	0 (0%)
*Curtobacterium flaccumfaciens*	1 (0.3%)	0 (0%)	1 (2.8%)	0 (0%)
*Glutamicibacter* spp.	1 (0.3%)	1 (0.3%)	0 (0%)	0 (0%)
*Lactococcus lactis*	1 (0.3%)	0 (0%)	1 (2.8%)	0 (0%)
*Lelliottia amnigena*	1 (0.3%)	1 (0.3%)	0 (0%)	0 (0%)
*Leuconostoc mesenteroides*	1 (0.3%)	1 (0.3%)	0 (0%)	0 (0%)
Total	400	355	36	9

Note. CM = clinical mastitis; NaS = Non-aureus staphylococci; *E. coli* = *Escherichia coli*. Data are presented as absolute numbers with percentages in parentheses. Percentages in the “Total frequency, n (%)” column are calculated based on the total number of samples (n = 400). Percentages in the columns “no CM”, “non-severe CM” and “severe CM” are calculated within each respective clinical group.

## Data Availability

The raw data supporting the conclusions of this study are available from the corresponding author upon reasonable request. Due to privacy and data protection restrictions concerning the participating farms, the data are not publicly available.

## Abbreviations

The following abbreviations are used in this manuscript:

CM	clinical mastitis
RTT	reticuloruminal temperature
AI	artificial intelligence
LPS	Lipopolysaccharide
*E. coli*	*Escherichia coli*
LTA	Lipoteichoic acid
PPV	positive predictive value
NSAID	non-steroidal anti-inflammatory drug
BC	bacteriological cure
SCC	somatic cell count
NaS	non-aureus staphylococci
